# Astronauts well-being and possibly anti-aging improved during long-duration spaceflight

**DOI:** 10.1038/s41598-021-94478-w

**Published:** 2021-07-21

**Authors:** Kuniaki Otsuka, Germaine Cornelissen, Satoshi Furukawa, Yutaka Kubo, Koichi Shibata, Koh Mizuno, Hiroshi Ohshima, Chiaki Mukai

**Affiliations:** 1 Executive Medical Center, Totsuka Royal Clinic, Tokyo Women’s Medical University, Related Medical Facility, Sinjuku City, Tokyo Japan; 2 Halberg Chronobiology Center, University of Minnesota, Minneapolis, MN USA; 3 Space Biomedical Research Group, Japan Aerospace Exploration Agency, Ibaraki, Japan; 4 Department of Medicine, Medical Center East, Tokyo Women’s Medical University, Tokyo, Japan; 5 Faculty of Education, Tohoku Fukushi University, Miyagi, Japan; 6 Tokyo University of Science, Tokyo, Japan

**Keywords:** Neuroscience, Physiology, Climate sciences, Environmental sciences, Planetary science, Cardiology, Health care, Medical research, Astronomy and planetary science, Engineering

## Abstract

This study assesses how circadian rhythms of heart rate (HR), HR variability (HRV) and activity change during long-term missions in space and how they relate to sleep quality. Ambulatory 48-h ECG and 96-h actigraphy were performed four times on ten healthy astronauts (44.7 ± 6.9 years; 9 men): 120.4 ± 43.7 days (Before) launch; 21.1 ± 2.5 days (ISS01) and 143.0 ± 27.1 days (ISS02) after launch; and 86.6 ± 40.6 days (After) return to Earth. Sleep quality was determined by sleep-related changes in activity, RR-intervals, HRV HF- and VLF-components and LF-band. The circadian amplitude of HR (HR-A) was larger in space (ISS01: 12.54, *P* = 0.0099; ISS02: 12.77, *P* = 0.0364) than on Earth (Before: 10.90; After: 10.55 bpm). Sleep duration in space (ISS01/ISS02) increased in 3 (Group A, from 370.7 to 388.0/413.0 min) and decreased in 7 (Group B, from 454.0 to 408.9/381.6 min) astronauts. Sleep quality improved in Group B from 7.07 to 8.36 (ISS01) and 9.36 (ISS02, *P* = 0.0001). Sleep-related parasympathetic activity increased from 55.2% to 74.8% (pNN50, *P* = 0.0010) (ISS02). HR-A correlated with the 24-h (r = 0.8110, *P* = 0.0044), 12-h (r = 0.6963, *P* = 0.0253), and 48-h (r = 0.6921, *P* = 0.0266) amplitudes of the magnetic declination index. These findings suggest associations of mission duration with increased well-being and anti-aging benefitting from magnetic fluctuations.

## Introduction

Previous investigations reported that circadian rhythm disruption and sleep problems, with impact on human aging, are pervasive among astronauts^1–7^. Nevertheless, several recent investigations provide evidence of anti-aging effects of long-duration space travel. In the National Aeronautics and Space Administration (NASA) Twin Study, the identical twin astronaut monitored before, during, and after a 1-year mission onboard the ISS had lengthened telomeres as compared to his twin serving as a genetically matched ground control^8^. Because telomere length is considered a marker of cellular aging, aging being usually associated with decreased telomere length^9,10^, the NASA twin study suggests a possible anti-aging effect of long-duration space travel. Another study on blood DNA methylation of six participants of the Mars-500 mission, a high-fidelity 520-day ground simulation experiment, showed that mission duration was associated with significant decreases in epigenetic aging at the 168- and 300-day time points^11^. DNA methylation, including DNAmPhenoAge, is a robust predictor of mortality risk that is also highly correlated with chronological age^11,12^.

Other investigations also showed anti-aging or even longevity effects of space travel, such as the significantly extended lifespan of *Caenorhabditis elegans* after a 9-day spaceflight^13^, and of *Drosophila melanogaster* after a 13-day spaceflight^14^. Our previous study also showed anti-aging effects of 6-month space flights, which were associated with improved HRV reflecting good health and anti-aging in seven astronauts^15^. Surprisingly, we found that magnetic fluctuations affect the brain’s default mode network (DMN) activity by stimulating the VLF component of HRV in a light-dependent manner and/or with help from the circadian clock.

Human aging on Earth is also generally associated with a weakening of the circadian system and a decrease in sleep efficiency. Earlier, based on analysis of ECG records of 7 astronauts during long-duration missions on the ISS, we observed an improvement in SDANN, Triangular Index, and TF after 4.5 months in space^15^. We then hypothesized that these results could indicate an anti-aging effect. Herein, we revisit our earlier finding based on data from 10 astronauts, and investigate possible mechanisms underlying anti-aging effects of long-duration space travel, focusing on the circadian system. We assess the circadian variation of heart rate (HR) and endpoints of heart rate variability (HRV) and estimate sleep efficiency based on 48-h ambulatory ECG records complemented by 96-h actigraphy. We examine (1) how circadian rhythms change in space, a larger 24-h amplitude assumed to reflect a stronger circadian system; and (2) how spaceflight affects sleep duration, symptoms of insomnia, and sleep quality. In order to understand how the observed changes may have occurred, we study (3) how spaceflight affects parasympathetic nerve activity, and (4) how magnetic fluctuations in space affect circadian behavior.

## Subjects and methods

### Subjects

Ten healthy astronauts (9 men, 1 woman) participated in this ISS (International Space Station) JAXA (Japan Aerospace Exploration Agency) investigation, named “Biological Rhythms 48 Hrs”. Their mean (± SD) age was 44.7 ± 6.9 years. Their mean stay in space was 155.7 ± 26.0 days. Astronauts had passed class III physical examinations from the National Aeronautics and Space Administration (NASA). The study was approved by the Institutional Review Boards of NASA, ESA (European Space Agency) and JAXA. Informed consent was obtained from all participants. A detailed explanation of the study protocol was given to the astronauts before they gave written, informed consent, according to the Declaration of Helsinki Principles. All methods were performed in accordance with the JAXA/ESA/NASA guidelines and regulations.

Universal Time Coordinated (UTC) is used aboard the ISS. The windows are covered during night hours to give the impression of darkness because the station experiences 16 sunrises and sunsets per day. Astronauts follow a strict 24-h routine, waking up at 06:00 and retiring for sleep at 21:30^16^. At the time of data collection of this study, artificial lighting from both incandescent and fluorescent light sources was used on the ISS; maximal light intensity was 700 lx^17^.

### Experimental protocols

Around-the-clock ambulatory 48-h ECG records were obtained by using a two-channel Holter recorder (FM-180; Fukuda Denshi). Measurements were made four times. First (Before), recording occurred 120.4 ± 43.7 (mean ± SD; range: 50–183) days before launch, except for one astronaut who, due to technical problems, was recorded on day 469 after return to Earth. Two recordings were obtained on the ISS, early (ISS01, 21.1 ± 2.5, 18–26 days after launch), and late (ISS02, 143.0 ± 27.1, 103–183 days after launch) during their mission. The last recording (After) was obtained 86.6 ± 40.6 (17–156) days after return to Earth. In one astronaut, actigraphy could not be performed during ISS02, and in two astronauts, ambulatory 48-h ECG records were not obtained after return to Earth, because of a recording failure due to electrodes’ poor contact to the skin.

The 48-h ECG records were subdivided into two 24-h spans to assess sleep duration and quality of sleep, and to compare HRV endpoints between days of relatively higher or lower magnetic activity.

Astronauts were classified by their sleep duration in space: in Group A (n = 3), sleep duration lengthened in space during both ISS01 and ISS02 compared to Before, and in Group B (n = 7), sleep duration was shorter during both ISS01 and IDD02 than Before.

### Analysis of heart rate variability

Data collection and measurement procedures were conducted as previously reported^15,18–21^. Briefly, for HRV measurements, RR intervals between normal QRS waveforms were extracted as normal-to-normal (NN) intervals. Time-domain HRV indices, including r-MSSD and pNN50, and frequency-domain measures, including conventional VLF- (0.003–0.04 Hz), LF- (0.04–0.15 Hz), and HF- (0.15–0.40 Hz) components^22^, as well as LF-band (0.01–0.05 Hz), MF1-band (0.05–0.10 Hz), MF2-band (0.10–0.15 Hz), and HF-band (0.15–0.20 Hz), reflecting an activation of the default mode network (DMN)^21^ were obtained with the MemCalc/CHIRAM (Suwa Trust GMS, Tokyo, Japan) software^23^.

### Actigraphy

A 96-h actigraphy record started 48 h before the start of 48-h ECG monitoring. Actigraphy was shown to be highly correlated with polysomnographically-defined sleep timing, even under spaceflight conditions^5,6,24^. Astronauts wore wrist-borne actiwatches (Actiwatch Spectrum; Phillips/Respironics, Murrysville, PA, USA) on the nondominant wrist, providing sleep/wake activity in 1-min epochs.

### Cosine curve fitting for estimating period, amplitude and acrophase by cosinor

The circadian period of activity and ECG-derived measures was determined by the Maximum Entropy Method (MEM)^23^. Single 24-h, 12-h or 48-h cosine curves were fitted independently to the data by cosinor^25–27^ to estimate their respective amplitude and acrophase in addition to the MESOR (Midline Estimating Statistic Of Rhythm, a rhythm-adjusted mean). Changes in circadian amplitude assessed the response in circadian rhythmicity to the space environment.

### Determining sleep duration, and scoring sleep quality and symptoms of insomnia

**Figure 1:**
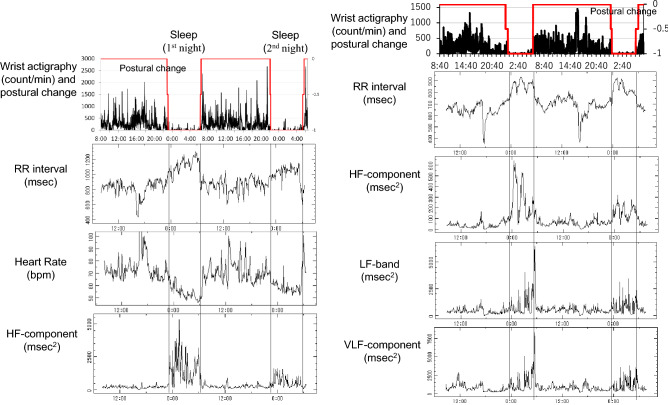
Estimation of sleep span (left) and assessment of sleep quality (right). Left: Sleep span is estimated by wrist activity data with postural change (row 1), RR-intervals (row 2), heart rate (row 3), and changes in the HF-component (row 4). This example shows day-to-day differences in the sleep-related increase in the HF-component of HRV. The spectral power of the HF-component is higher on the first day (row 4). In addition, the nocturnal HR dip is blunted on the second day as compared to the first day (row 3). Right: Representative example of a 48-h concomitant record of wrist activity, RR-intervals, and HF-component, LF-band and VLF-component of HRV. Right and left results are from different astronauts. Sleep quality is assessed by determining whether there is a sleep-related (1) decrease in activity (row 1); and increase during the sleep span in (2) RR-intervals (row 2); (3) power in the HF-component (row 3); (4) power of the LF-band (row 4); and (5) power of the VLF-component (row 5). A sleep quality score is obtained by summing the 5 items for each night, on a scale of 0 to 10 points (Yes/No is 1/0).

Sleep at night was estimated by actigraphy, RR-intervals, heart rate and changes in the HF-component of HRV, as shown in the left panel of Fig. 1. Sleep duration was determined as the average between two consecutive sleep spans. An “insomnia score” between 0 and 10 was determined based on four items. A score of 3, 2, or 1 was assigned if the average sleep duration was below 4.5 h, between 4.5 and 6 h, or between 6 and 7.5 h, respectively. A score of 3, 2, or 1 was assigned if the average sleep latency was more than 30 min, between 20 and 30 min, or between 10 and 20 min, respectively. A score of 1 was assigned if sleep was interrupted. A score of 3, 2, or 1 was assigned if the time of awakening was earlier than usual on Earth by more than 60 min, 40 to 60 min, or 20 to 40 min, respectively. The insomnia score was obtained by summing the scores from the four items.

Sleep quality was estimated by using not only the actigraphy data and RR-intervals, but also the spectral power of the HF-component, LF-band and VLF-component of HRV, as shown in the right panel of Fig. 1, because these indices reflect the quality of rest, activity of the DMN, and fluctuations that are important for health, respectively. Five items contributed to determining the sleep quality score. Each item had a 2-point scale (0 to 1 for each of two consecutive nights, corresponding to an answer of “No” or “Yes” to each question item). Summing the scores from the five items yielded a score on a scale of 0 to 10, a higher score meaning a better sleep quality. The five items consist of whether there is a sleep-related (1) decrease in activity; and increase during sleep in (2) RR-intervals; (3) power in the HF-component; (4) power of the LF-band; and (5) power of the VLF-component. In the example shown in the right panel of Fig. 1, a sleep-related increase in RR-intervals was prominent during sleep on both nights (counted as 1 point each). Similarly, sleep-related increases in the power of the HF-component, LF-band, and VLF-component were also observed on both nights, as was a sleep-related decrease in activity. Accordingly, the sleep quality score in this case is 10.

### Assessment of parasympathetic activity by nocturnal HR dip and sleep-related HRV rise in space

A blunted HR dip during sleep has been associated with all-cause mortality^28^. HR is expected to decrease by at least 10% during sleep as compared to the awake span. Non-dipping was defined as a decrease of less than 10%. HRV is also known to increase during sleep. The nocturnal rise was calculated by comparison with the awake span. Changes in sleep-related HR-dipping and HRV-rising during spaceflight are assessed.

### Assessment of magnetic fluctuations

The ISS is protected from the space environment by Earth's magnetic field. The ISS orbits the Earth every 90 min at an altitude of 330 to 480 km. As we reported previously^15^, we used geomagnetic measurements at 1-min intervals available from the Auroral Observatory of the University of Tromsø, Norway (69°39′ N, 18°56′ E). The indices considered herein are total intensity (F, in nT), declination (D, angle between geographic and magnetic north, in degrees), inclination (I, angle between horizontal plane and magnetic direction, in degrees), horizontal intensity (H, in nT), and vertical intensity (Z, in nT).

Historically, the estimation of the ionospheric electric field used ionospheric currents and field-aligned currents from ground magnetic records, along with incoherent scatter radars, satellite measurements of X-ray and UV aurorae^29^. This type of study over the past two hundred years about the structure and temporal changes of the Sun–Earth space, now called space weather, has become of great interest to space science^30–32^.

### Effects of magnetic fluctuations on the circadian amplitude of HR in space

Correlations between the circadian amplitude of HR and the 12-, 24-, or 48-h amplitudes of geomagnetic indices were estimated using Pearson’s correlation coefficient.

### Statistical analyses

Data were expressed as mean ± standard deviation (SD). Space-related changes in HRV endpoints were assessed by computing differences between their values assumed during ISS01 and/or ISS02 and those obtained before launch (Before), analyzed by the paired t-test. Effects of magnetic activity on HRV endpoints were also compared to before launch (Before) by the paired t-test, using the Stat Flex (Ver. 6) software (Artec Co., Ltd., Osaka, Japan). *P*-values less than 0.05 were considered to indicate statistical significance.

## Results

### Circadian rhythm of sleep–wake cycle and heart rate during spaceflight

Actigraphy records spanned on average 5453.5 ± 828.5 min (Before), 6565.6 ± 1270.1 min (ISS01), 6761.0 ± 529.0 min (ISS02) and 5273.7 ± 1290.0 min (After). The corresponding estimates of the circadian period were 23.55-h (Before), 24.32-h (ISS01), 23.66-h (ISS02) and 23.91-h (After). Estimates of the acrophase at a 24-h trial period were 14:32 (Before), 14:15 (ISS01), 14:12 (ISS02) and 14:13 (After). Circadian periods and 24-h acrophases did not change statistically significantly in space. The circadian amplitude of activity did not change with statistical significance either. The circadian sleep–wake cycle was resilient to the mission environment in space (Table 1).

**Table 1:** Circadian period of the sleep–wake cycle, heart rate, and intrinsic cardiovascular regulatory system.

Actigraphy	Before (n = 10)		ISS01 (n = 10)		ISS02 (n = 9)		After (n = 10)	
	Mean	SD	Mean	SD	Mean	SD	Mean	SD
Period (h)	23.55	1.47	24.32	0.98	23.66	0.56	23.91	1.39
Amplitude (N/min)	152.92	50.20	145.88	42.66	144.58	44.83	138.63	79.46
Acrophse (hh:mm)	14:32	1:01	14:15	1:08	14:12	1:18	14:13	1:23
MESOR (N/min)	194.9	60.0	166.5	40.4	162.2*	39.7	168.7	72.6

Heart rate	Before (n = 10)		ISS01 (n = 10)		ISS02 (n = 10)		After (n = 8)	
	Mean	SD	Mean	SD	Mean	SD	Mean	SD
Period (h)	23.52	1.99	24.15	1.85	23.33	1.91	25.11	2.28
Amplitude (bpm)	10.90	1.79	12.54^##^	2.78	12.77^#^	2.69	10.55	3.49
Acrophse (hh:mm)	14:39	1:07	15:04	1:19	14:13	1:33	15:40	2:12
MESOR (bpm)	69.32	6.84	71.53	6.60	73.94*	6.11	70.26	10.20

*P < 0.05 vs. Before.

^#^*P* < 0.05 vs. After, ^##^*P* < 0.01 vs. After.

*P*-values not corrected for multiple testing.

**Figure 2:**
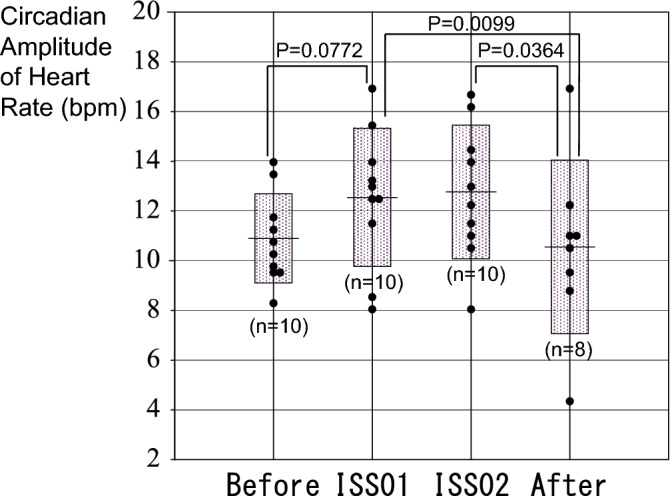
Change in the circadian amplitude of HR related to the space mission. The circadian amplitude of HR increased during spaceflight from 10.90 ± 1.79 bpm (Before) to 12.54 ± 2.78 (ISS01), 12.77 ± 2.69 (ISS02), returning to 10.55 ± 3.49 (After).

The circadian amplitude of HR increased during spaceflight from 10.90 bpm (Before) to 12.54 (ISS01) and 12.77 (ISS02), returning to 10.55 (After), Fig. 2. There were no statistically significant changes in the circadian period or acrophase of HR in space. Such an amplification of the circadian variation in HR differs from results of previous investigations^2–7^.

The 48-h and 3.5-day amplitudes of activity tended to be reduced in space. The 48-h amplitude dropped from 39.85 ± 30.14 (Before) to 22.12 ± 16.05 (ISS01) (*P* = 0.1041) and 27.63 ± 17.93 (ISS02), and the 84-h amplitude dropped from 44.51 ± 37.22 (Before) to 18.57 ± 16.36 (ISS01) (*P* = 0.0588) and 22.64 ± 14.46 (ISS02). Similarly, the 48-h amplitude of HR was reduced in space from 3.78 ± 1.55 (Before) to 2.23 ± 9.90 (ISS01, *P* = 0.0426) and 2.91 ± 1.30 (ISS02). These results reinforce the higher prominence of the circadian rhythm of these variables in space.

### Sleep duration and scoring of sleep quality and symptoms of insomnia

**Figure 3:**
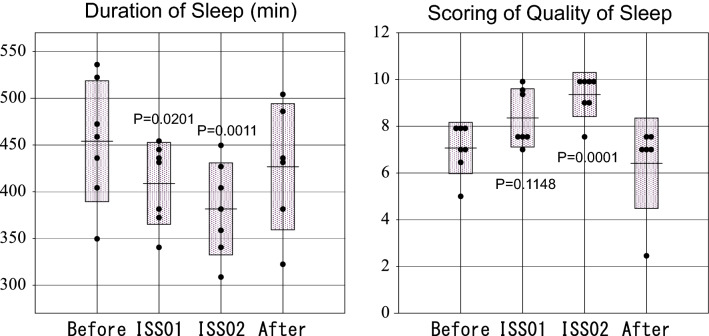
Changes in sleep duration (left) and quality of sleep score (right) in Group B astronauts. Sleep duration shortened from 454.0 ± 64.7 min (Before) to 408.9 ± 43.8 min (ISS01, *P* = 0.0201) and 381.6 ± 49.2 min (ISS02, *P* = 0.0011) during spaceflight, returning to 426.7 ± 67.5 min after the mission (left). Quality of sleep improved from 7.07 ± 1.10 to 8.36 ± 1.25 (ISS01, *P* = 0.1148) and 9.36 ± 0.95 (ISS02, *P* = 0.0001), returning to 6.42 ± 1.93 after return to Earth (right).

In group A, insomnia and quality of sleep scores did not statistically significantly change in space, as compared to pre-launch, Table 2. By definition, astronauts in group B shortened their sleep duration in space, which changed from 454.0 min (Before) to 408.9 min (ISS01, *P* = 0.0201) and 381.6 min (ISS02, *P* = 0.0011), returning to 426.7 min (After), Fig. 3 (left). The quality of sleep score improved from 7.07 (Before) to 8.36 (ISS01), and 9.36 (ISS02, *P* = 0.0001), returning to 6.42 (After), Fig. 3 (right). No statistically significant change was noted for the insomnia score. The change in sleep duration may reflect a regression toward the mean as sleep duration before launch was shorter in Group A than in Group B (370.7 vs. 454.0 min, *P* = 0.0984). Sleep duration no longer differed in space between the two groups (ISS01: 388.0 vs. 408.9 min, *P* = 0.5549; ISS02: 413.0 vs. 381.6 min, *P* = 0.3720) or after return to Earth (474.5 vs. 426.7 min, *P* = 0.3791). Sleep quality did not differ either between the two groups, before launch (7.83 vs. 7.07, *P* = 0.2836), in space (ISS01: 9.00 vs. 8.36, *P* = 0.4257; ISS02: 7.83 vs. 9.36, *P* = 0.2680) or after return to Earth (6.25 vs. 6.42, *P* = 0.9120).

**Table 2:** Change in sleep duration, insomnia, and quality of sleep scores during spaceflight.

Group A (n = 3)	Before (n = 3)		ISS01 (n = 3)		ISS02 (n = 3)		After (n = 2)	
	Mean	SD	Mean	SD	Mean	SD	Mean	SD
Sleep duration (min)	370.7	64.4	388.0	62.2	413.0	44.9	474.5	9.2
Insomnia score	2.50	2.18	2.33	1.89	1.83	1.16	2.00	0.00
Quality of sleep score	7.83	0.29	9.00*	0.50	7.83	3.33	6.25	0.35

Group B (n = 7)	Before (n = 7)		ISS01 (n = 7)		ISS02 (n = 7)		After (n = 6)	
	Mean	SD	Mean	SD	Mean	SD	Mean	SD
Sleep duration (min)	454.0	64.7	408.9*	43.8	381.6**	49.2	426.7	67.5
Insomnia score	2.10	0.89	2.07	0.45	2.71	1.19	2.50	1.34
Quality of sleep score	7.07	1.10	8.36	1.25	9.36**	0.95	6.42	1.93

Paired t-test; *P < 0.05, **P < 0.01 (vs. Before, same Group).

### Nocturnal HR dip and HRV rise during long-duration spaceflight

As shown in Table 3, HR dropped by more than 10% during sleep in both groups on the ISS. In Group B, the HR dip became deeper during ISS01 (*P* = 0.0375) and ISS02 (*P* = 0.0375) as compared to pre-launch. The nocturnal rise increased during ISS02 in r-MSSD (*P* = 0.0492) and pNN50 (*P* = 0.0010), HRV indices reflecting parasympathetic activity. The nocturnal dip also decreased reciprocally in LF/HF (*P* = 0.0161), which reflects sympathetic activity. During ISS02, the nocturnal rise also increased in the MF1-band reflecting DMN activity (*P* = 0.0070), and in the VLF-component showing well-being (*P* = 0.0303), Table 3.

**Table 3:** Sleep-related changes in HR dip and HRV rise in space.

Group A	Before			ISS01			ISS02			After		
	n	Mean	SD	n	Mean	SD	n	Mean	SD	n	Mean	SD
**Dipping ratio**												
Heart rate	4	24.3	3.3	6	25.1	3.9	6	23.5	5.3	4	23.9	4.2
LF/HF ratio	4	51.2	13.6	6	35.6	25.8	6	36.2	33.4	4	33.2*	12.7
**Rising ratio**												
r-MSSD	4	34.5	8.8	6	34.8	15.5	6	32.1	24.9	4	20.0*	14.4
pNN50	4	60.8	20.1	6	53.4	17.9	6	− 7.7	103.2	4	40.1	11.4
HF-component (0.15–0.40 Hz)	4	57.0	11.8	6	54.0	18.5	6	41.7	47.1	4	33.1**	16.6
MF1-band (0.05–0.10 Hz)	4	9.7	17.7	6	36.4**	10.1	6	26.6	21.4	4	2.2	15.0
VLF-component (0.003–0.04 Hz)	4	43.7	13.8	6	41.9	14.7	6	36.7	31.6	4	43.5	12.8

Group B	Before			ISS01			ISS02			After		
	n	Mean	SD	n	Mean	SD	n	Mean	SD	n	Mean	SD
**Dipping ratio**												
Heart rate	14	20.9	4.1	14	24.1*	5.3	14	24.8*	5.0	12	20.4	5.2
LF/HF ratio	14	42.1	28.0	14	32.6	24.3	14	27.2*	33.4	12	22.8	48.7
**Rising ratio**												
r-MSSD	14	25.1	21.9	14	26.2	23.8	14	37.3*	8.8	12	33.5	16.8
pNN50	14	55.2	16.9	14	59.9	30.2	14	74.8**	10.8	12	59.7	19.0
HF-component (0.15–0.40 Hz)	14	50.5	23.5	14	44.0	23.3	14	51.8	13.0	12	43.3	34.1
MF1-band (0.05–0.10 Hz)	14	6.9	38.3	14	17.5	30.3	14	31.1**	27.8	12	17.4	31.8
VLF-component (0.003–0.04 Hz)	14	34.1	22.4	14	36.6	19.7	14	49.8*	14.3	12	37.4	12.0

Paired t-test *P < 0.05, **P < 0.01 (versus Pre-launch); Ratios estimated during each night separately.

*P*-values not corrected for multiple testing.

In Group A, the data were insufficient to estimate the drop/rise ratio in 1 astronaut before launch and in another astronaut after return to Earth. Similarly in Group B, the data were insufficient to estimate the drop/rise ratio in 1 astronaut after return to Earth. Data analyzed on separate 24-h days.

### Effects of magnetic fluctuations on circadian HR amplitude in space

**Figure 4:**
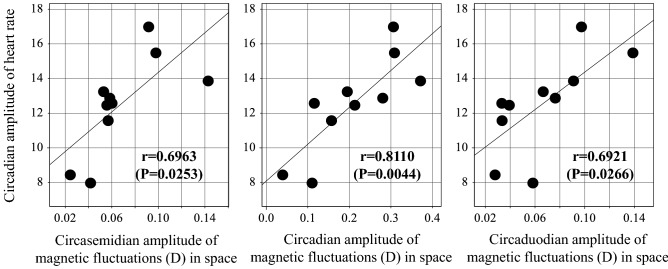
Representative example of correlation between the circadian amplitude of HR and the amplitude of the 12-, 24- and 48-h components of the geomagnetic declination (D) of the geomagnetic field. Effect of magnetic fluctuations on the circadian amplitude of human HR is suggested by its strong correlation with the 12-h (circasemidian; r = 0.6963, *P* = 0.0253, left panel), circadian (r = 0.8110, *P* = 0.0044, middle panel), and 48-h (circaduodian; r = 0.6921, *P* = 0.0266, right panel) amplitudes of D, the declination of the geomagnetic field.

MEM spectra of geomagnetic indices during spans matching the 48-h ECG records showed not only a peak around one cycle in 24 h, but also peaks around one cycle in 48 h and around one cycle in 12 h, corresponding to the circaduodian and circasemidian components, respectively. The circadian amplitude of HR correlated statistically significantly with the circadian (r = 0.8110, *P* = 0.0044), circasemidian (r = 0.6963, *P* = 0.0253) and circaduodian (r = 0.6921, *P* = 0.0266) amplitudes of the geomagnetic declination index, Fig. 4. Among geomagnetic indices, the geomagnetic declination index showed the strongest association, as shown in Table 4. The circasemidian amplitude of the geomagnetic declination index also correlated statistically significantly with the circadian amplitude of the VLF-component (r = 0.6963, *P* = 0.0253), MF1-band (r = 0.7398, *P* = 0.0144), and HF-band (r = 0.7702, *P* = 0.0091), Table 4. Its circadian amplitude also correlated with the circadian amplitude of the VLF-component (r = 0.7383, *P* = 0.0148). An effect of magnetic fluctuations was not found to affect the circadian acrophase of HR or any of the HRV endpoints.

**Table 4:** Effects of 24-, 12-, and 48-h geomagnetic fluctuations in space on the circadian amplitude of HR and HRV.

Effects of circadian amplitude of magnetic fluctuations in space (n = 10)	HR (circadian amplitude) during ISS01			VLF-component (circadian amplitude) during ISS01			MF1-band (circadian amplitude) during ISS01			HF-band (circadian amplitude) during ISS01		
	r	t-value	*P*-value	r	t-value	*P*-value	r	t-value	*P*-value	r	t-value	*P*-value
Declination	0.8110	3.9215	**0.0044**	0.7383	3.0958	**0.0148**	0.4319	1.3545	0.2126	0.5269	1.7535	0.1176
Horizontal component	0.7016	2.7849	**0.0237**	0.4639	1.4810	0.1769	0.3474	1.0480	0.3253	0.4080	1.2641	0.2418
Vertical component	0.4107	1.2739	0.2385	− 0.0810	− 0.2299	0.8239	− 0.0467	− 0.1323	0.8980	− 0.1348	− 0.3848	0.7104
Inclination	0.6945	2.7304	**0.0258**	0.4211	1.3132	0.2255	0.3340	1.0022	0.3456	0.3904	1.1994	0.2647
Total field intensity	0.2844	0.8391	0.4258	0.3217	0.9609	0.3647	− 0.2405	− 0.7009	0.5032	− 0.2617	− 0.7671	0.4651

Effects of circasemidian amplitude of magnetic fluctuations in space (n = 10)	HR (circadian amplitude) during ISS01			VLF (circadian amplitude) during ISS01			MF1 (circadian amplitude) during ISS01			HF-band (circadian amplitude) during ISS01		
	r	t-value	*P*-value	r	t-value	*P*-value	r	t-value	*P*-value	r	t-value	*P*-value
Declination	0.6963	2.7440	**0.0253**	0.6963	2.7439	**0.0253**	0.7398	3.1104	**0.0144**	0.7702	3.4156	**0.0091**
Horizontal component	0.5709	1.9667	0.0848	0.7041	2.8047	**0.0230**	0.1038	0.2952	0.7754	0.2134	0.6179	0.5538
Vertical component	0.3265	0.9769	0.3572	0.3225	0.9638	0.3634	0.4432	1.3984	0.1995	0.4348	1.3656	0.2092
Inclination	0.5587	1.9052	0.0932	0.6798	2.6220	**0.0306**	0.1567	0.4487	0.6656	0.2545	0.7443	0.4780
Total field intensity	0.4228	1.3195	0.2235	0.3849	1.1794	0.2721	0.4608	1.4687	0.1801	0.4700	1.5060	0.1705

Effects of circaduodian amplitude of magnetic fluctuations in space (n = 10)	HR (circadian amplitude) during ISS01			VLF (circadian amplitude) during ISS01			MF1 (circadian amplitude) during ISS01			HF-band (circadian amplitude) during ISS01		
	r	t-value	*P*-value	r	t-value	*P*-value	r	t-value	*P*-value	r	t-value	*P*-value
Declination	0.6921	2.7120	**0.0266**	0.4783	1.5404	0.1620	0.2050	0.5924	0.5699	0.2551	0.7462	0.4769
Horizontal component	0.6137	2.1983	0.0592	0.2878	0.8499	0.4201	0.0618	0.1751	0.8653	0.0415	0.1174	0.9094
Vertical component	0.4474	1.4149	0.1948	− 0.1252	− 0.3568	0.7305	− 0.0830	− 0.2356	0.8197	− 0.1355	− 0.3869	0.7089
Inclination	0.5951	2.0946	0.0695	0.2443	0.7125	0.4964	0.0493	0.1395	0.8925	0.0384	0.1088	0.9161
Total field intensity	0.4919	1.5979	0.1487	− 0.2185	− 0.6333	0.5442	− 0.1677	− 0.4812	0.6433	− 0.3110	− 0.9255	0.3818

*P-*values not corrected for multiple testing. Bold *P*-values indicates statistically significant correlation of HRV endpoint with geomagnetic index.

## Discussion

The most striking result of this investigation is the larger prominence of the circadian HR rhythm in space in ten astronauts living on the ISS for about 6 months, Fig. 2, an indication that long-duration spaceflight may have slowed down aging. These results contrast with earlier reports by others^2–7^. Most of these studies, however, were conducted during shorter missions on the MIR station or on shuttle missions. Daily schedules may not have been as regimented as in current ISS conditions, and no information on the lighting conditions were provided^2–6^. Results from the only study that was conducted on the ISS during long-duration missions similar to ours showed that sleep duration was shorter and of poorer quality when crewmembers’ sleep episodes were misaligned relative to the estimated endogenous circadian temperature minimum^7^. The large circadian amplitude of HR and of some of the HRV endpoints may be an indication that in our study circadian misalignment may have minimal, although we did not measure temperature. Another aspect of our results, which was not examined in these earlier studies and may deserve further investigation is our finding that both the 3.5-day component of activity and the 48-h component of HR weakened in space, supporting an enhancement of the circadian rhythm.

Misalignment of the circadian clock could impair the health of astronauts. Evidence from genetic animal models and from humans under circadian misalignment (such as shift work) shows that disrupting the coordination between the endogenous clock and physiological processes can seriously affect health. Circadian disruption can lead to cardiovascular, metabolic and psychiatric disorders, cancer and cognitive decline in the aged. The International Agency for Research on Cancer (IARC), part of the World Health Organization (WHO), enlisted circadian disruption as a probable human carcinogenic (Group 2A) in 2007^33–44^. Conversely, enforcement of the circadian rhythm impairs cancer progression and increases quality of life^41^. In patients with lung and breast cancer, the presence of a circadian rhythm is a survival predictor^45,46^; patients with colorectal cancer who have a robust circadian rhythm survive longer and have a better quality of life than patients who have a misaligned circadian system and poor sleep^47,48^. Thus, enhancement of the circadian HR rhythm in space, observed herein, may be associated with well-being and reflect a heightened health status. Whether the amplified circadian HR rhythm stems from the regular daily routine on the ISS or from exposure to the space environment itself remains to be determined. Below, we review how long-duration space travel might promote anti-aging.

Among the several reasons why the circadian rhythm of HR strengthened in space, the astronauts’ strictly regulated scheduled day on the ISS comes to mind first. Because the body has peripheral clocks located in each organ (heart, lung, liver, muscles, kidneys, retina, etc.) that optimize the function of each organ according to the environmental context, allowing adaptation of the organism to environmental changes, the circadian clock network has an adaptive function^49,50^. The astronauts’ day starts by awakening at 06:00, followed by post-sleep activities and a morning inspection of the station. The crew then eats breakfast and takes part in a daily planning conference with Mission Control before starting work at around 08:10. The first scheduled exercise of the day follows, after which the crew continues work until 13:05. Following a one-hour lunch break, the afternoon consists of more exercise and work before astronauts carry out their pre-sleep activities beginning at 19:30, including dinner and a crew conference. The scheduled sleep span begins at 21:30. Such a regular life schedule, in particular, a regular light/dark cycle, regular arousal stimuli, regular food intake, and in principle regularly programmed exercise can thus synchronize the circadian system of astronauts^51,52^.

In addition, space-environmental stimuli, including magnetic fluctuations and microgravity can be involved in the amplification of the circadian rhythm of HR. The role of magnetic fluctuations in circadian clock function, how sleep enhances parasympathetic tone, and how the DMN is activated, gauged by HRV behavior in the VLF, MF1- and HF-bands, are additional insights obtained from the present investigation (Table 4). A recent study^53^ found that both the amplitude and period of the endogenous circadian oscillation are affected by magnetic stimulation, and that the effects are strongly circadian stage-dependent. Applied at the appropriate phase, magnetic stimulation enhances the circadian amplitude of bioluminescence of cultured suprachiasmatic nucleus slices.

Microgravity can also induce an over 3-fold increase in the circadian amplitude of clock genes^54–57^. In addition to light, gravity is another synchronizer affecting circadian patterns with significant changes of general behavior, hormone synthesis, body temperature and metabolism^54^.

### Sleep in space

Sleep duration was shorter in space than on Earth in 7 of the 10 astronauts (Group B). On average, they slept 6 h and 49 min during ISS01 and 6 h 22 min during ISS02, which represents 90.1% and 84.1% of their sleep duration before launch, respectively; after return to Earth, they slept 7 h 7 min (94.0% of pre-launch sleep duration), Fig. 3 (left) and Table 2. In the other 3 astronauts (Group A), sleep duration during ISS01 and ISS02 was 6 h and 28 min and 6 h and 53 min, respectively (Table 2).

Sleep duration is associated with several health and functional consequences, with both short (≤ 6 h) and long (≥ 9 h) sleep duration increasing the risk of negative outcomes^58–64^. Several studies have shown that short sleep duration, as well as long sleep duration, impacts inflammation^65^. In the population-based InCHIANTI study (*n* = 751), concentrations of TNF and CRP were increased at extremes of both short and long sleep. Sleep duration between 6 and 7 h observed during space travel suggests that it is sufficient for attenuating inflammatory disease risk at the systemic, cellular, and genomic levels, with implications for inflammaging and molecular processes of aging, both in Group A and B astronauts, as Irwin and Opp proposed^65^.

Insomnia independently contributes to the risk of inflammaging processes of aging^66,67^. The insomnia score of astronauts of both Groups A and B was kept well between 1.83 and 2.71 during space travel (Table 2).

The quality of sleep score of astronauts improved in space, another sign that aging may have slowed down on the ISS. In astronauts of Group A, the increase was mostly noted during ISS01 (from 7.83 to 9.00, *P* = 0.0198), whereas in astronauts of Group B, the sleep score increased from 7.07 before launch to 8.36 during ISS01 and to 9.36 during ISS02 (*P* = 0.0001), Table 2 and Fig. 3 (right). Sleep quality is a complex construct that is quite difficult to evaluate with sufficient precision^68–70^. We estimated quality of sleep based on actigraphy and on the circadian profiles of several HRV endpoints (Fig. 1). Among them, the HF-component is known to express quality of sleep^22,71^; the LF-band is thought to reflect an activation of the temporoparietal junction (TPJ), a central member of hubs of the DMN, serving a broader adaptive purpose^21,72,73^; the VLF-component reflects fluctuations that are fundamental to health and well-being, because the heart itself intrinsically generates the VLF-component of HRV^15,74–76^. Since these endpoints were used to derive the quality of sleep score, and since they are associated with health preservation, we can consider that a high quality of sleep score is indicative of health.

We found that sleep quality was improved in space, contrary to previous investigations. Numerous studies have linked sleep to the circadian rhythm, also in humans^42,77,78^. Magnetic stimulation in space may be another contributory factor. Several previous investigations indeed demonstrated that magnetic stimulation at night induced deep sleep slow waves^79,80^.

### Parasympathetic activity assessed by sleep-related HR dip and HRV rise in space

Sleep-related parasympathetic activity improved in astronauts of Group B, reaching values similar to those of astronauts in Group A. The nocturnal rise in HRV, reflecting parasympathetic activity, increased in space during ISS02 as compared to pre-flight: r-MSSD increased from 25.1 to 37.3% (*P* = 0.0492) and pNN50 from 55.2% to 74.8% (*P* = 0.0010). These responses in space agree with work by Gundel et al., who suggested that the parasympathetic drive to the heart during sleep may be stronger in space than on Earth^81^. Reciprocally, LF/HF, reflecting sympathetic activity, decreased in space compared to pre-flight (from 42.1% to 27.2% during ISS02, *P* = 0.0161). Hence, the nocturnal HR dip was more pronounced in space than before launch: it increased from 20.9% to 24.1% (ISS01, *P* = 0.0375) and 24.8% (ISS02, *P* = 0.0384), returning to pre-launch values after return to Earth (20.4%), Table 3. In addition, we found that the MF1-band, reflecting DMN activity, and the VLF-component, fundamental to health and well-being, increased during ISS02 as compared to pre-flight (MF1: from 6.9% to 31.1.%, *P* = 0.0070; VLF: from 34.1% to 49.8%, *P* = 0.0303), Table 3.

Since a blunted nocturnal decrease in HR (by less than 10%) has been associated with all-cause mortality^82–84^, responses observed in HR and HRV indices may suggest a positive physiological adaptation to microgravity in space.

### Effects of magnetic fluctuations on the circadian amplitude of HR

Earlier, we reported a possible anti-aging effect of magnetic fluctuations in space, as gauged by HRV endpoints of astronauts during long-duration missions on the ISS^15^. Herein, we confirm this result by our comparison of several HRV endpoints between the day of higher versus the day of lower magnetic activity. In astronauts of Group B (n = 7) during ISS01, days of higher magnetic activity are associated with a higher MESOR of the HF-component (164.2 ± 84.5 vs. 145.3 ± 85.6, *P* = 0.0381), of pNN50 (7.2 ± 6.0 vs. 5.9 ± 5.9, *P* = 0.0361), of the VLF-component (2644.7 ± 1151.3 vs. 2292.7 ± 1016.3, *P* = 0.0040), and of the LF-band (1663.1 ± 786.8 vs. 1482.5 ± 724.3, *P* = 0.0450). During ISS02, the power of the HF-component is larger and the nocturnal HR dip is more pronounced on the day of higher magnetic activity, as illustrated for one case in Fig. 1 (left).

The circadian amplitude of HR correlated positively with magnetic disturbances in space along the scales of 24 h (r = 0.8110, *P* = 0.0044), 12 h (r = 0.6963, *P* = 0.0253) and 48 h (r = 0.6921, *P* = 0.0266), Table 4 and Fig. 4. As life depends on processes fluctuating at many different frequencies, many showing co-periodisms with the broad environment, it is not surprising that several components of space weather are associated with an amplification of the circadian HR rhythm in space. Invisible fluctuating cosmic factors (such as solar activity and magnetic disturbances) can affect virtually every cell and electrical circuits in biological systems^85–87^. Although the underlying mechanisms are far from being fully understood, our previous studies based on 7-day/24-h ECG found a significant graded decrease in HRV endpoints in response to geomagnetic storms. Based on these results, we postulated the existence of a light-dependent human magnetoreception system^88,89^ and proposed that human cryptochrome-2 can act as a magnetic sensor^90^.

As humans respond to space weather, Halberg et al.^87^ proposed that broad time structures (chronomes) in humans resonate with cycles found in the environment. The study of these effects is part of chronomics medicine, including chronoastrobiology^27,87^, the science studying effects of heliogeomagnetics on biota. On Earth, circaseptans in human HR were amplified during spans when they were detected in solar activity^86^. Herein, we showed that magnetic fluctuations also had a distinct impact on the circadian rhythm of important cardiovascular functions in space.

### Limitations

This is the first report showing that magnetic activity can reinforce the circadian system in space. The study is limited, however, by the relatively short ECG records of 48 h, monitored only twice during a 6-month space mission. Longer ECG records are desirable as shown by our previous 7-day/24-h investigations on Earth^27,85,88,89^.

Another limitation is the lack of brain oscillatory activity data, as discussed in our previous investigations^15,21^, although a number of studies showed that HRV is like a mirror reflecting functions of the neural network^72,91,92^. However, these associations are extremely complex^93–95^, and we need future investigations to directly depict the brain oscillatory activity in space.

Future studies would benefit from including measures of telomere length and DNA methylation, which are generally accepted indicators of anti-aging, as a complement to the increased prominence of circadian rhythms and the increase in sleep quality documented herein.

Fully understanding physiology in space is challenging in view of the multiple factors involved beyond weightlessness. Fluid redistribution, isolation, and the changed work environment are elements that all contribute to the astronauts' well-being and physiological adaptation to space. Results herein have shed light on the merit of distinguishing between different stages of adaptation to the space environment, realizing that some mechanisms involved may take several months. Much work remains to be done, however, before arriving at unambiguous and reliable answers to the multi-faceted questions that long-duration spaceflights entail.

## Conclusion

Herein, we provided evidence that in space the circadian rhythm of HR was strengthened, sleep quality was improved, and parasympathetic tone was enhanced at night, by taking advantage of magnetic fluctuations and by activating DMN activities. Our previous investigation also showed that magnetic fluctuations in the magnetosphere can affect and enhance HRV endpoints in space, thereby perhaps conveying an anti-aging effect, probably in association with the brain DMN, in a light-dependent manner and/or with help from the circadian clock^15^. These findings suggest associations of long-duration space travel with increased well-being and anti-aging properties.

Such information could enable the formulation of novel hypotheses, or account for unanticipated results, for instance from data collected during a magnetic storm that we now know can affect the heart and brain. Thus, magnetic disturbances have become a concern in programs of space exploration, even though further studies on the molecular mechanisms underlying the action of the electromagnetic fields, in combination with microgravity, are warranted.

## Data Availability

Restrictions from Japan’s Aerospace Exploration Agency apply to the availability of the data supporting the findings of this study. which were used under license for the current study. As such, they are not publicly available.
